# Pattern Formation through Temporal Fractional Derivatives

**DOI:** 10.1038/s41598-018-23470-8

**Published:** 2018-03-22

**Authors:** Hongwei Yin, Xiaoqing Wen

**Affiliations:** 1 School of Science, Nanchang University, Nanchang, 330031 P.R. China; 2 Numerical Simulation and High-Performance Computing Laboratory, Nanchang University, Nanchang, 330031 P.R. China

## Abstract

It is well known that temporal first-derivative reaction-diffusion systems can produce various fascinating Turing patterns. However, it has been found that many physical, chemical and biological systems are well described by temporal fractional-derivative reaction-diffusion equations. Naturally arises an issue whether and how spatial patterns form for such a kind of systems. To address this issue clearly, we consider a classical prey-predator diffusive model with the Holling II functional response, where temporal fractional derivatives are introduced according to the memory character of prey’s and predator’s behaviors. In this paper, we show that this fractional-derivative system can form steadily spatial patterns even though its first-derivative counterpart can’t exhibit any steady pattern. This result implies that the temporal fractional derivatives can induce spatial patterns, which enriches the current mechanisms of pattern formation.

## Introduction

As a kind of organized heterogeneous macroscopic structure, spatial patterns exist extensively in the natural world ranging from chemical reaction systems to physical systems and to ecological systems. In order to interpret the formation of the patterns observed in his experiments^1^, Alan Turing proposed a reaction-diffusion model (currently popularly called as the Turing model). By model analysis, he showed that if the underlying system undergoes Turing bifurcation, then a so-called Turing pattern (i.e., a spontaneously-organized spatial heterogeneous pattern away from the stable equilibriums of the system) can occur. This pioneer work of Turing not only came into being theoretical foundation for understanding diverse patterns occurring in the natural world, but also opened a new research direction-pattern dynamics that have received extensive attention and are currently still a hot topic in many scientific fields, such as molecular biology^2–4^, biochemistry^5^, development biology^6^, epidemiology^7,8^, mechanics^9^, and so on.

In spite of extensive applications, Turing bifurcation theory is limited in nonlinear reaction-diffusion systems with the temporal first derivative. However, many realistic processes are well described by nonlinear reaction-diffusion equations with the temporal fractional derivatives because this class of derivative can deal comfortably with memory effect in dynamical systems^10,11^. In fact, more and more fractional-derivative differential equations have been successfully used in biological materials^12^, fluid mechanics^13^, quantum mechanics^14^, and so on. Thereinto, spatial patterns for some fractional-derivative reaction-diffusion systems, whose first-derivative counterparts can produce some spatial patterns, were deeply discussed in^15–20^. The current results for such a kind of systems mainly discovered how the temporal and spatial fractional derivatives change transient dynamical behaviors and affect structure of spatial patterns. These results imply that the nonlinearity still plays the main role in the formation of spatial patterns. Therefore, immediately arises a question whether or not there is a certain causal relationship between the fractional derivative and Turing instability, that is, for a temporal fractional-derivative reaction-diffusion system whose temporal first-derivative counterpart cannot form any spatial pattern, can the temporal fractional derivative induce the Turing instability and produce spatial patterns? To answer this question, we consider a classical diffusive prey-predator system with the Holling II functional response, where the temporal fractional derivative is introduced into this system because of the memory for the prey’s and predator’s behaviors, as follows:

1a $$\frac{{\partial }^{\eta }u}{{\partial }^{\eta }t}=\Delta u+u(1-u)-\frac{uv}{u+\alpha },$$

1b $$\frac{{\partial }^{\eta }v}{{\partial }^{\eta }t}=d\Delta v+\frac{\beta uv}{u+\alpha }-\gamma v,$$

where *u*(*x*, *t*) and *v*(*x*, *t*) respectively describe the prey’s and predator’s densities at time *t* on the spatial position *x*. *α* is a prey’s density at which the predator has the maximum kill rate; *β* and *γ* are the maximum birth rate and dead rate of the predator, respectively; The operator ∆ can describe the diffusion of these two species from the higher density region to the lower. $d$ denotes diffusion rate of the predator and is scaled with respect to the prey’s diffusion. The parameters α, β and γ are positive. $\frac{{\partial }^{\eta }u}{{\partial }^{\eta }t}\,\,$with *η ∈ *(0, 1) is the standard Caputo’s partial derivative with respect to the time variable *t*, which can describe the memory character of the prey’s and predator’s dynamical behaviors. In particular, when *η = *1 the system (1) degenerates into the temporal first-derivative system, which represents the instantaneous behaviors of the prey and predator. For this case, the system (1) cannot product any spatial pattern, see ref.^21^. However, for the system (1) with *η ∈ *(0, 1), it can be found that the temporal fractional derivatives can induce Turing instability and that steady-state spatial patterns form ultimately for the system (1). This result, which enriches the current mechanisms of forming spatial patterns, is our main contribution in this paper.

The article is organized as follows: in Sec. 2, we first study the stability of the corresponding ODE system of the system (1). In Sec. 3, we derive the condition inducing the Turing instability for the system (1) and give out some numerical simulations of spatial patterns. In the final section, a brief discussion and conclusive remark are given.

## Methods

### Dynamic properties of the fractional-order ODE system

We first examine the stability and Hopf bifurcation for the ordinary differential equation (ODE) version of the system (1). Omitting the diffusion terms in the system (1), one has the following ODE system

2a $$\frac{{d}^{\eta }u}{{d}^{\eta }t}=u(1-u)-\frac{uv}{u+\alpha },$$

2b $$\frac{{d}^{\eta }v}{{d}^{\eta }t}=\frac{\beta uv}{u+\alpha }-\gamma v,$$

The system (2) always has the equilibrium points ${E}_{0}=(0,0)$ and ${E}_{1}=(1,0)$. Besides, if

3 $${\rm{\beta }} > \gamma ,0 < \alpha  < \frac{\beta -\gamma }{\gamma },$$

there exists another positive equilibrium point ${E}_{2}=({u}^{\ast },{v}^{\ast })$, where ${u}^{\ast }=\frac{\alpha \gamma }{\beta -\gamma }$ and ${v}^{\ast }=(1-{u}^{\ast })({u}^{\ast }+\alpha )$. The Jacobian matrix of the system (2) at ${E}_{2}$ is

4 $${J}_{2}=[\begin{array}{cc}\frac{\alpha \gamma }{\beta }(\frac{1}{\alpha }-\frac{\beta +\gamma }{\beta -\gamma }) & -\frac{\gamma }{\beta }\\ -(\alpha +1)\gamma +\beta  & 0\end{array}]$$

and its characteristic equation is

5 $$\,{\lambda }^{2}-{A}_{1}\lambda +{B}_{1}=0,$$

where ${A}_{1}=\frac{\alpha \gamma }{\beta }(\frac{1}{\alpha }-\frac{\beta +\gamma }{\beta -\gamma })$ and ${B}_{1}=\frac{\gamma (\beta -\gamma -\alpha \gamma )}{\beta }$. For *η* = 1, *E*_2_ is stable when the parameters *α* and *β* are fixed in the set ${{\rm{\Pi }}}_{1}$ defined as

6 $${{\rm{\Pi }}}_{1}=\{(\alpha ,\beta ,\gamma )\in {R}_{+}^{3}|\beta  > \gamma ,\frac{\beta -\gamma }{\beta +\gamma } < \alpha  < \frac{\beta -\gamma }{\gamma }\}.$$

However, for $\eta \in (0,1)$, the stability of *E*_2_ is more complex and depends on the inequality

7 $$|\text{arg}\lambda | > \frac{\eta \pi }{2}$$

for the roots *λ* in the equation (5)^22^. For convenience, we consider the two cases:

A. the two roots *λ* in (5) have negative real parts;
B. these two roots are true complex numbers and have positive real parts.

For the case **A**, the stability of *E*_2_ is easy to be identified by the relationship between the roots and

coefficients in (5). For the case **B**, we assume the roots in (5) $\lambda =P\pm iQ,P,Q\in {R}_{+}$ and can obtain the equivalent inequality of (7),

8 $$\tan (\text{arg}\lambda )=\frac{Q}{P}=\frac{\sqrt{4{B}_{1}-{A}_{1}^{2}}}{{A}_{1}} > \,\tan (\frac{\eta \pi }{2}).$$

We define the parameter set

9 $${{\rm{\Pi }}}_{2}=\{(\alpha ,\beta ,\gamma ,\eta )\in {R}_{+}^{3}\times (0,1)|\frac{\sqrt{4{B}_{1}-{A}_{1}^{2}}}{{A}_{1}} > \,\tan (\frac{\eta \pi }{2})\}.$$

Then, *E*_2_ is stable if the parameters *α* and *β* are in the set Π_1_ or in the set Π_2_.

**Figure 1:**
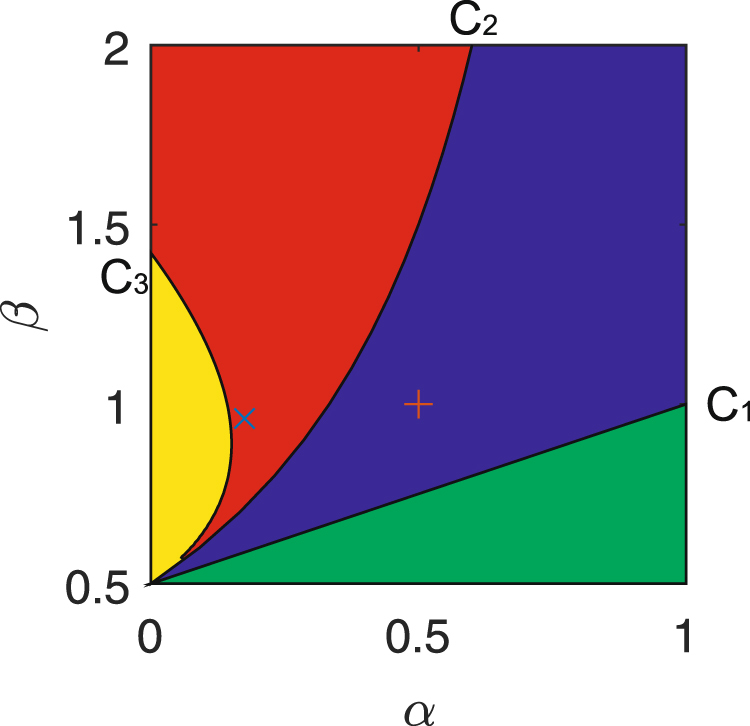
The bifurcation diagram of the parameters *α* and *β* for the system (2) with *η* = 0.8 and 1. Therein, *γ* = 0.5, the position of the mark “×” is (0.175, 0.95) and the one of “+” is (0.5, 1).

**Figure 2:**
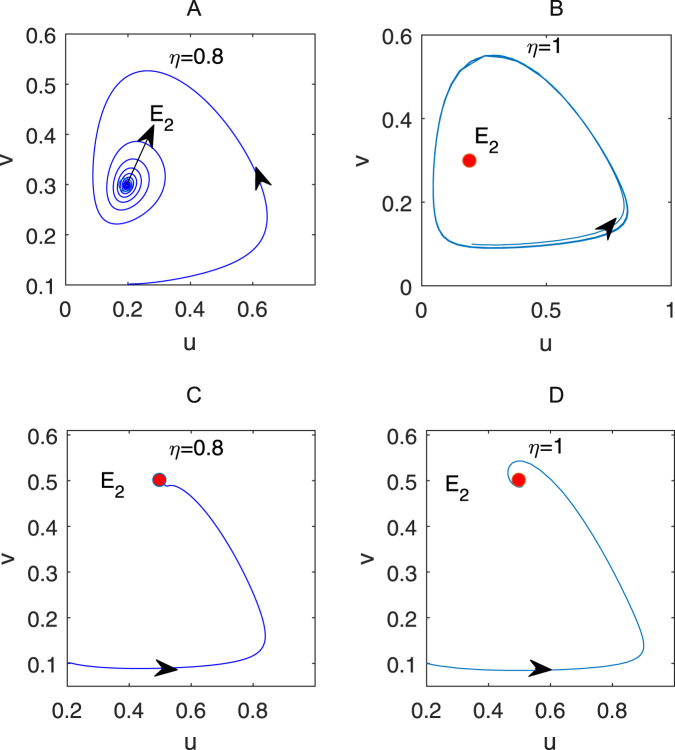
The dynamical trajectories of the fractional- and first-derivative systems. (**A**,**B**) with the parameters *α* = 0.175 and *β* = 0.95; (**C**,**D**) with the parameters *α* = 0.5 and *β* =1. All of these four trajectories start from *u*_0_ = 0.2 and *v*_0_ = 0.1. The other parameters are taken as in Fig. 1.

The parameter set Π_2_ seems complex but nonempty, for example, we take *γ* = 0.5 and *η* = 0.8 and plot a parameter *α − β* bifurcation diagram, see Fig. 1. In Fig. 1, the curve *C*_1_ coming from the equation $\alpha =\frac{\beta -\gamma }{\gamma }$ is a transcritical bifurcation one, the curve *C*_2_ coming from the equation $\alpha =\frac{\beta -\gamma }{\beta +\gamma }$ is a Hopf bifurcation one for the system (2) with *η* = 1, and the curve *C*_3_ coming from the equation $\frac{\sqrt{4{B}_{1}-{A}_{1}^{2}}}{{A}_{1}}=\,\tan (\frac{\eta \pi }{2})$ is a Hopf bifurcation one for the system (2) with *η* = 0.8. For the temporal first-derivative system (2), *E*_2_ is stable when the parameters *α* and *β* are taken in the blue region of Fig. 1. However, for the system (2) with *η* = 0.8, if *α* and *β* are taken in the red or blue region, then *E*_2_ is also stable. To illustrate this result, we choose two groups of the parameters in the form of (*α*, *β*): (0.175, 0.95) (marked as “×”) in the red region and (0.5, 1) (marked as “+”) in the blue region of Fig. 1, and respectively plot the dynamical trajectories of the systems (2) with *η* = 0.8 and 1 in Fig. 2. In Fig. 2A, *E*_2_ is stable for the system (2) with *η* = 0.8 for the parameters *α* = 0.175 and *β* = 0.95; for the system (2) with the same parameters *α* and *β* but *η* = 1, the periodic trajectory around *E*_2_ appears in Fig. 2B. For the parameters *α* = 0.5 and *β* = 1, both of the fractional- and first-system (2) show the stability of *E*_2_ in Fig. 2C and Fig. 2D. These results imply that the temporal fractional derivatives can enlarge the range of the parameters such that *E*_2_ keeps stable, as is well known to us.

### Turing patterns

We will mainly show the main result of this paper that the temporal fractional derivative can form steady-state spatial patterns for the system (1) in one- and two- dimension spatial regions. Firstly, we shall discuss Turing instability for the system (1).

### Turing instability

The necessary condition of the Turing instability, it is well known, is that an equilibrium point for an ordinary different equation system is stable, but unstable for its corresponding reaction-diffusion system. Here, we only consider a small spatiotemporally inhomogeneous perturbation near *E*_2_ in the form

10 $${(u,v)}^{T}={({u}^{\ast },{v}^{\ast })}^{T}+{\epsilon }\exp (\lambda t+ikr)$$

where *λ* is the growth of the perturbation; *k* is a wave number vector; *I* is the imaginary unit; *r* is the spatial vector with the dimension number 1, 2 or 3; ${\epsilon }$ is a small parameter and represents the perturbation strength. Substituting (10) into (1) and only keeping the first-power term of the parameter ${\epsilon }$, we can obtain the Jacobin matrix

11 $$J(k)=[\begin{array}{ll}\frac{\alpha \gamma }{\beta }(\frac{1}{\alpha }-\frac{\beta +\gamma }{\beta -\gamma })-{k}^{2} & -\frac{\gamma }{\beta }\\ -(\alpha +1)\gamma +\beta  & -d{k}^{2}\end{array}].$$

Its characteristic equation is written into

12 $$\,{\lambda }^{2}-{A}_{2}(k)\lambda +{B}_{2}(k)=0,$$

where

13a $${A}_{2}(k)=-(1+d){k}^{2}+\frac{\alpha \gamma }{\beta }(\frac{1}{\alpha }-\frac{\beta +\gamma }{\beta -\gamma }),$$

and

13b $${B}_{2}(k)=d{k}^{4}-\frac{d\alpha \gamma }{\beta }(\frac{1}{\alpha }-\frac{\beta +\gamma }{\beta -\gamma }){k}^{2}+\frac{\gamma (\beta -\gamma -\alpha \gamma )}{\beta }.$$

From the characteristic equation (12) and the discussion on the stability of the system (2) in the above Section, *E*_2_ for the first-derivative system (2) with the parameters in Π_1_ is always stable for any wave numbers *k*. Consequently, the Turing instability does not occur for the first-derivative system (2) and any steady-state spatial pattern cannot appear.

Next, we shall focus on the instability of *E*_2_ due to some wave numbers *k* ≠ 0 for the fractional-derivative system (1), but *E*_2_ is stable for the fractional-derivative system (2). From the equations (5) and (12), we find that *E*_2_ might become unstable for the fractional- derivative system (1) with the parameters *α*, *β* and the fractional derivative *η* in the set Π_2_. From the explicit expression of *B*_2_(*k*) and through some tedious calculation, we can obtain a parameter set Π_3_ of *E*_2_’s instability for the system (1), which is defined by

14 $${{\rm{\Pi }}}_{3}=\{(\alpha ,\beta ,\gamma ,d)\in {R}_{+}^{4}|\sqrt{\frac{\beta (\beta -\gamma -\alpha \gamma )}{d\gamma }}+\frac{\alpha (\beta +\gamma )}{2(\beta -\gamma )} < \frac{1}{2}\}.$$

**Figure 3:**
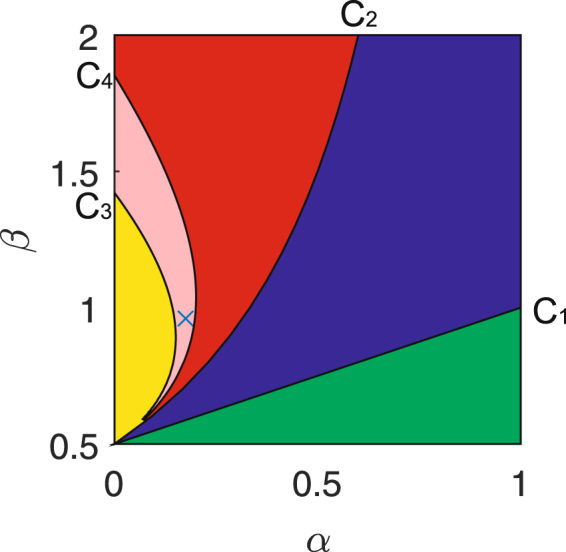
The parameter set of the Turing instability for the fractional-derivative system (1) labeled in pink. Herein, *d* = 20 and the other parameters as in Fig. 1. The curve *C*_4_ is a Turing bifurcation one, and the position marked by “×” is (0.175, 0.95).

For the fractional-derivative system (1), the Turing instability arises when the parameters and fractional derivative are taken in the intersection Π = Π_2_ ∩ Π_3_. Therefore, the set Π is called as a Turing instability parameter set. In fact, the set Π is still nonempty, for example, take *d* = 20 and the other parameters as in Fig. 1, and plot the Turing instability parameter set (labeled by the pink color) in Fig. 3, where the curve *C*_4_ is a Turing bifurcation one and comes from the equation

15 $$\sqrt{\frac{\beta (\beta -\gamma -\alpha \gamma )}{d\gamma }}+\frac{\alpha (\beta +\gamma )}{2(\beta -\gamma )}=\frac{1}{2}.$$

**Figure 4:**
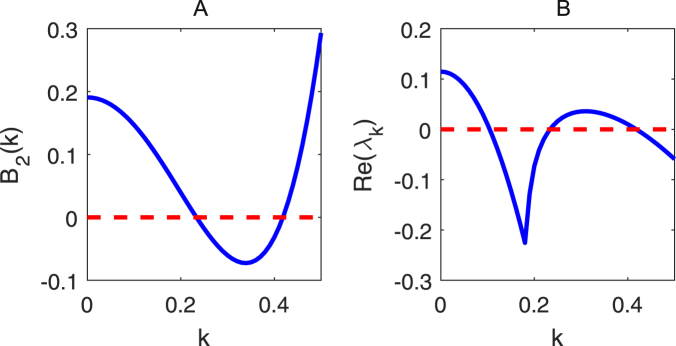
The dispersion coefficient and the maximal real part of the roots in (7). The parameters are taken as *α*  =  0.175, *β = *0.95, *γ = *0.5, *η = *0.8 and *d = *20.

Further, we choose the parameters *α* = 0.175, *β* = 0.95, *γ* = 0.5, *η* = 0.8 and *d* = 20, whose position (marked as “×”) is in the pink region of Fig. 3, and respectively plot the suitable dispersion coefficient in Fig. 4A and the real part of the characteristic roots in Fig. 4B against the wave numbers *k*. We find the coefficient *B*_2_(*k*) < 0 and the real part *Re*(*λ*_*k*_) < 0 for some wave numbers *k*, which indicates that the Turing instability occurs for the parameters and fractional derivative taken in the set Π.

## Results

### Turing patterns and numerical results

We simulate the system (1) in a bounded spatial domain with zero-flux boundary conditions, i.e., $\frac{\partial u}{\partial n}=\frac{\partial v}{\partial n}=0$ ($n$ denotes the normal unit vector of the boundary curve), which means that the prey and predator are isolated in the domain and absence of external in- put. To solve the system (1), we transform the continuous system to a finite dimensional system, i.e., discrete the system in both time and space. Without loss of generality, we take random perturbations around *E*_2_ as initial conditions of the system (1) in the form of ${u}_{0}={u}^{\ast }+\sigma {\xi }_{1}(x)$ and ${v}_{0}={v}^{\ast }+\sigma {\xi }_{2}(x)$, where *ξ*_1_(*x*) and *ξ*_2_(*x*) are stochastic processes with respect to the spatial variable *x*, obeying the uniform distribution on [0, 1] and mutually independent. The constant *σ* is noise strength.

**Figure 5:**
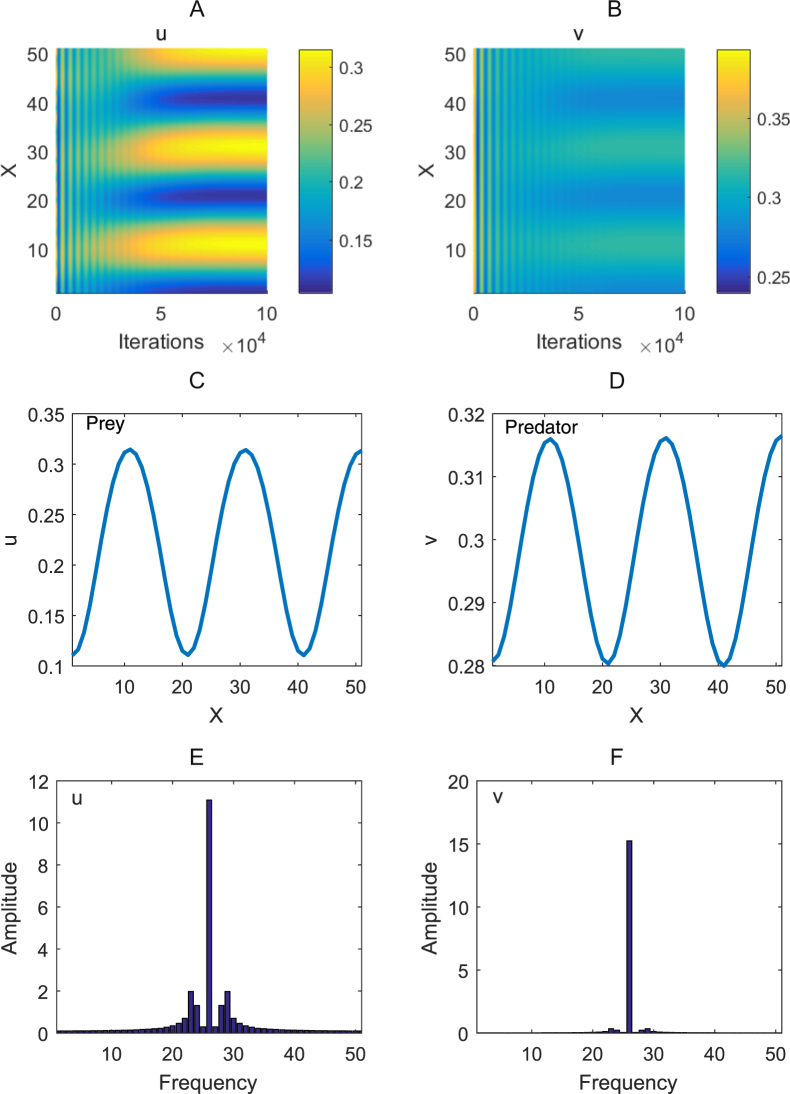
The dynamical behaviors of the prey and predator in the fractional-derivative system (1) with the one-dimension space and their steady-state spatial patterns. (**A** and **B**) are dynamical trajectories of the prey and predator, respectively. (**C** and **D**) respectively exhibit the steady-state spatial patterns of the prey and predator after 1 × 10^5^ iterations for the discrete time. (**E** and **F**) are the power spectrums for the prey’s and predator’s steady-state spatial patterns, respectively. Wherein, the parameters are taken as in Fig. 4.

We still take the parameters as *α = *0.175, *β* = 0.95, *γ* = 0.5, *η* = 0.8, *d* = 20 as well as *σ* = 0.1. By using the center difference method in space and the approximation of the Caputo’s derivative by the Grunwald-Letnikov one in time^23^, we numerically perform the fractional-derivative system (1) on the one-dimension domain with the spatial length *L* = 50, the time step ∆*t* = 5 × 10^*−*3^ and the uniform spatial step ∆*x = *1. The prey’s and predator’s dynamical behaviors are respectively depicted in Fig. 5A and B. After 1 × 10^5^ iterations for the discrete time, their dynamical trajectories converge to spatially inhomogeneous steady states. This implies that the steady-state spatial patterns of the prey and predator ultimately form for the fractional-derivative system (1), which is respectively shown in Fig. 5C and D. Figure 5E and F respectively exhibit the power spectrums of the prey’s and predator’s spatial patterns in the one-dimension domain. These two figures verify the formation of the spatially inhomogeneous steady state for the fractional-derivative system (1).

**Figure 6:**
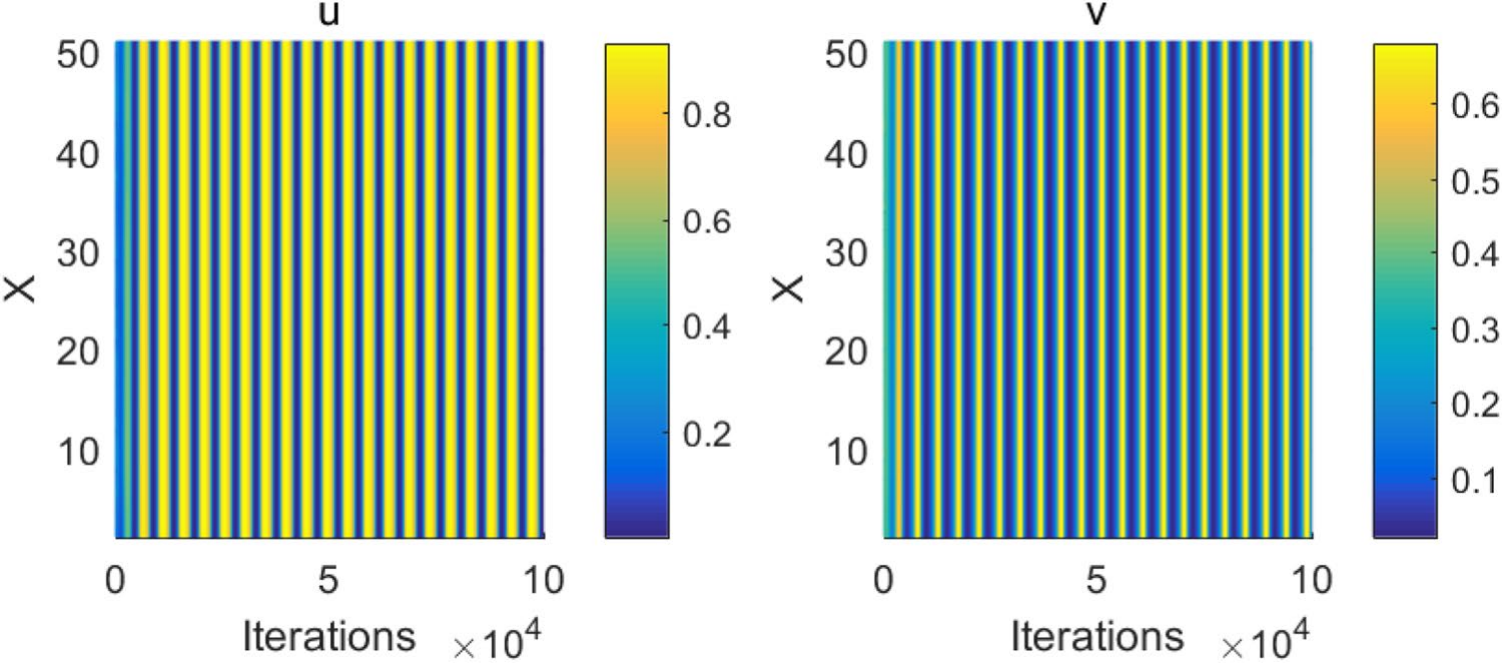
The spatially homogeneous periodic orbits of the prey and predator in the one-dimension space system (1) with the first derivative. The other parameters are taken as in Fig. 5.

**Figure 7:**
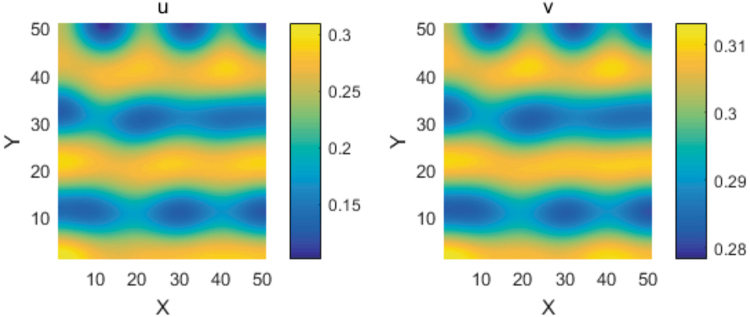
The spatial patterns of the prey and predator for the fractional-derivative system (1) in the two-dimension square domain. The parameters are taken as in Fig. 5.

By applying the same discrete scheme, the central difference method in space and the forward Euler method in time, and taking the same parameters and initial values, we numerically simulate the one-dimension space system (1) with the first derivative (i.e., *η* = 1), and yield the spatially homogeneous periodic solutions of the prey and predator in Fig. 6. The difference between Figs 5 and 6 with the same parameters implies that the fractional derivative can induce the Turing instability and product steady-state spatial patterns. Besides, by applying the same performing scheme and taking the same parameters as in Fig. 5, we simulate the fractional-derivative system (1) on the two-dimension square domain *L* × *L* = 50 × 50 with the uniform spatial step length ∆*x* = ∆*y* = 1 and the time step ∆*t* = 3 × 10^*−*3^. After 2 × 10^5^ iterations for the discrete time, the spatial two-dimension fractional-derivative system (1) ultimately converges to a steady state, and the prey’s and predator’s spatial patterns form, which are illustrated in Fig. 7.

**Figure 8:**
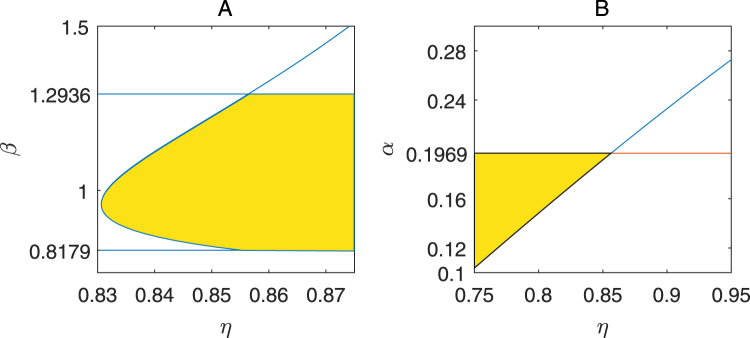
The Turing instability sets (labeled by yellow) of the parameters *β*, *α* and the fractional derivative *η*. (**A**) the Turing instability set of *β* and *η* with *α* = 0.175. (**B**) The Turing instability set of *α* and *η* with *β* = 0.95 in B. The other parameters are taken as in Fig. 5.

To uncover the effect of the fractional derivatives on the Turing instability for the system (1), we first fix *α = *0.175 to plot the Turing instability set of *β* and *η* in Fig. 8A, and then fix *β = *0.95 to plot the Turing instability set of *α* and *η* in Fig. 8B. Figure 8 demonstrates that the Turing instability for the system (1) depends on the fractional derivatives and the parameters in the system (1).

## Discussion

At present, some mechanisms of pattern formation have been uncovered, such as cross diffusion^24,25^, stochastic noise from external and internal environment^26–28^, nonlocal diffusion of single species^29,30^ and anomalous diffusion^15–20^. The current results imply that the nonlinear factor among these mechanisms, in essence, plays a main role in the pattern formation. Although some authors studied spatial patterns for some fractional- derivative systems, they only concerned how the fractional derivatives change spatial patterns of this kind of systems and neglected what role the fractional derivatives play in the pattern formation. In this paper, we studied the temporal fractional-derivative prey-predator diffusive system with the Holling II functional response whose corresponding temporal first- derivative system is impossible to form any spatial pattern, and showed that the fractional derivatives play a crucial role in forming the steadily spatial pattern. Our results make us reacquaint the work of the fractional derivatives on dynamical systems. Currently, various shapes of spatial patterns from diffusive systems have been clearly identified by the multi- scale method, such as dot pattern, strip pattern, spiral pattern and so on. It is necessary to use this method to further check the spatial patterns from the system (1). This problem is expecting a future study.
